# *Rickettsia* transmission from whitefly to plants benefits herbivore insects but is detrimental to fungal and viral pathogens

**DOI:** 10.1128/mbio.02448-23

**Published:** 2024-02-05

**Authors:** Pei-Qiong Shi, Lei Wang, Xin-Yi Chen, Kai Wang, Qing-Jun Wu, Ted C. J. Turlings, Peng-Jun Zhang, Bao-Li Qiu

**Affiliations:** 1 Engineering Research Center of Biotechnology for Active Substances, Ministry of Education, Chongqing Normal University, Chongqing, China; 2 Department of Computational Medicine and Bioinformatics, School of Medicine, University of Michigan, Ann Arbor, Michigan, USA; 3 Institute of Vegetables & Flowers, Chinese Academy of Agricultural Sciences, Beijing, China; 4 FARCE Laboratory, Institute of Biology, University of Neuchâtel, Neuchâtel, Switzerland; 5 College of Life and Environmental Sciences, Hangzhou Normal University, Huangzhou, China; Max Planck Institute for Chemical Ecology, Jena, Germany

**Keywords:** bacterial symbiont, *Rickettsia*, *Bemisia tabaci*, horizontal transmission, plant defense

## Abstract

Bacterial endosymbionts play important roles in the life histories of herbivorous insects by impacting their development, survival, reproduction, and stress tolerance. How endosymbionts may affect the interactions between plants and insect herbivores is still largely unclear. Here, we show that endosymbiotic *Rickettsia belli* can provide mutual benefits also outside of their hosts when the sap-sucking whitefly *Bemisia tabaci* transmits them to plants. This transmission facilitates the spread of *Rickettsia* but is shown to also enhance the performance of the whitefly and co-infesting caterpillars. In contrast, *Rickettsia* infection enhanced plant resistance to several pathogens. Inside the plants, *Rickettsia* triggers the expression of salicylic acid-related genes and the two pathogen-resistance genes *TGA 2.1* and *VRP*, whereas they repressed genes of the jasmonic acid pathway. Performance experiments using wild type and mutant tomato plants confirmed that *Rickettsia* enhances the plants’ suitability for insect herbivores but makes them more resistant to fungal and viral pathogens. Our results imply that endosymbiotic *Rickettsia* of phloem-feeding insects affects plant defenses in a manner that facilitates their spread and transmission. This novel insight into how insects can exploit endosymbionts to manipulate plant defenses also opens possibilities to interfere with their ability to do so as a crop protection strategy.

**IMPORTANCE:**

Most insects are associated with symbiotic bacteria in nature. These symbionts play important roles in the life histories of herbivorous insects by impacting their development, survival, reproduction as well as stress tolerance. *Rickettsia* is one important symbiont to the agricultural pest whitefly *Bemisia tabaci*. Here, for the first time, we revealed that the persistence of *Rickettsia* symbionts in tomato leaves significantly changed the defense pattern of tomato plants. These changes benefit both sap-feeding and leaf-chewing herbivore insects, such as increasing the fecundity of whitefly adults, enhancing the growth and development of the noctuid *Spodoptera litura*, but reducing the pathogenicity of *Verticillium* fungi and TYLCV virus to tomato plants distinctively. Our study unraveled a new horizon for the multiple interaction theories among plant-insect-bacterial symbionts.

## INTRODUCTION

In nature, approximately two-thirds of arthropods are associated with maternally inherited bacterial symbionts (1). These bacterial symbionts can be classified as primary symbionts such as *Portiera* in whiteflies, *Buchnera* in aphids, and *Carsonella* in psyllids, or secondary symbionts such as *Wolbachia*, *Rickettsia,* and *Hamiltonella* in many insects (2–5). Primary symbionts are obligate and provide nutrients that are lacking in unbalanced or restricted diets and are essential for the survival of their hosts (6). Secondary symbionts are facultative to their hosts and often manipulate their hosts’ reproduction (5, 7–10). They may also increase their hosts’ ability to resist important natural enemies, for example, endoparasitic wasps (11, 12) or pathogens (13). Finally, the symbionts may also increase their hosts’ tolerance to abiotic stress such as high temperatures (14).

Vertical transmission is the most frequent transmission mode for facultative endosymbionts of insects, but horizontal transmission also occurs (15–19). Bacterial endosymbionts like *Cardinium*, *Wolbachia,* and *Rickettsia* can be transferred from infected insects into plants, where other insects of the same or different species can acquire them through subsequent feeding on these plants (20–25).

In the case of *Rickettsia*, plants can serve as reservoirs for horizontal transmission of endosymbiotic *Rickettsia* among whiteflies. Caspi-Fluger et al. (21) and Li et al. (23) demonstrated that horizontal transmission of *Rickettsia* occurred between two different cryptic species of whitefly *Bemisia tabaci* (Gennadius), Middle East-Asia Minor 1 (MEAM1), and Mediterranean (MED), via cotton plants. These two species are biologically differentiated and reproductively isolated (26), which excludes the possibility that transmission occurs during mating.

The aforementioned studies have focused on the transmission routes and the direct effects of endosymbiont acquisition by herbivorous insects. However, they did not consider the possibility that, endosymbionts that are transmitted to plants may influence plant traits, such as growth and defense, and thereby indirectly influence the performance of the insects (19). We here specifically test if the exogenous introduction of a bacterial endosymbiont may trigger host plant responses that, in turn, may affect the performance of insects and pathogens on the same host plant.

We previously demonstrated that *Rickettsia belli* can infect all life stages of *B. tabaci*, including eggs, nymphs, and male and female adults (27). Importantly, Himler et al. (28) found that, compared to uninfected whiteflies, *Rickettsia-*infected whiteflies produce more offspring, have higher survival to adulthood, develop faster, and produce a higher proportion of daughters. However, the mechanism that mediates these benefits to the whitefly host has remained unknown. In the current study, we used a combination of molecular tools, biochemical analyses, and performance experiments to investigate how the horizontal transmission of *Rickettsia* endosymbionts from phloem-feeding whitefly adults into tomato plants affect insect and pathogen performance on these plants (Fig. S1). For the first time, our study has revealed the consequent effects of endosymbiont persistence in host plants. The transmission of *Rickettsia* into tomato plants makes the plants more suitable for their normal herbivorous host, the whitefly *B. tabaci*, as well as a chewing herbivore, the noctuid *S. litura* (Fabricius). In contrast, the *Rickettsia*-induced changes made the plants more resistant to a pathogenic fungus (*Verticillium dahliae* Klebahn), and two begomoviruses (Tomato yellow leaf curl virus, TYLCV; Papaya leaf curl China virus, PaLCuCNV) vectored by whiteflies.

## RESULTS

### The distribution and persistence of *Rickettsia* in tomato plants

**Fig 1:**
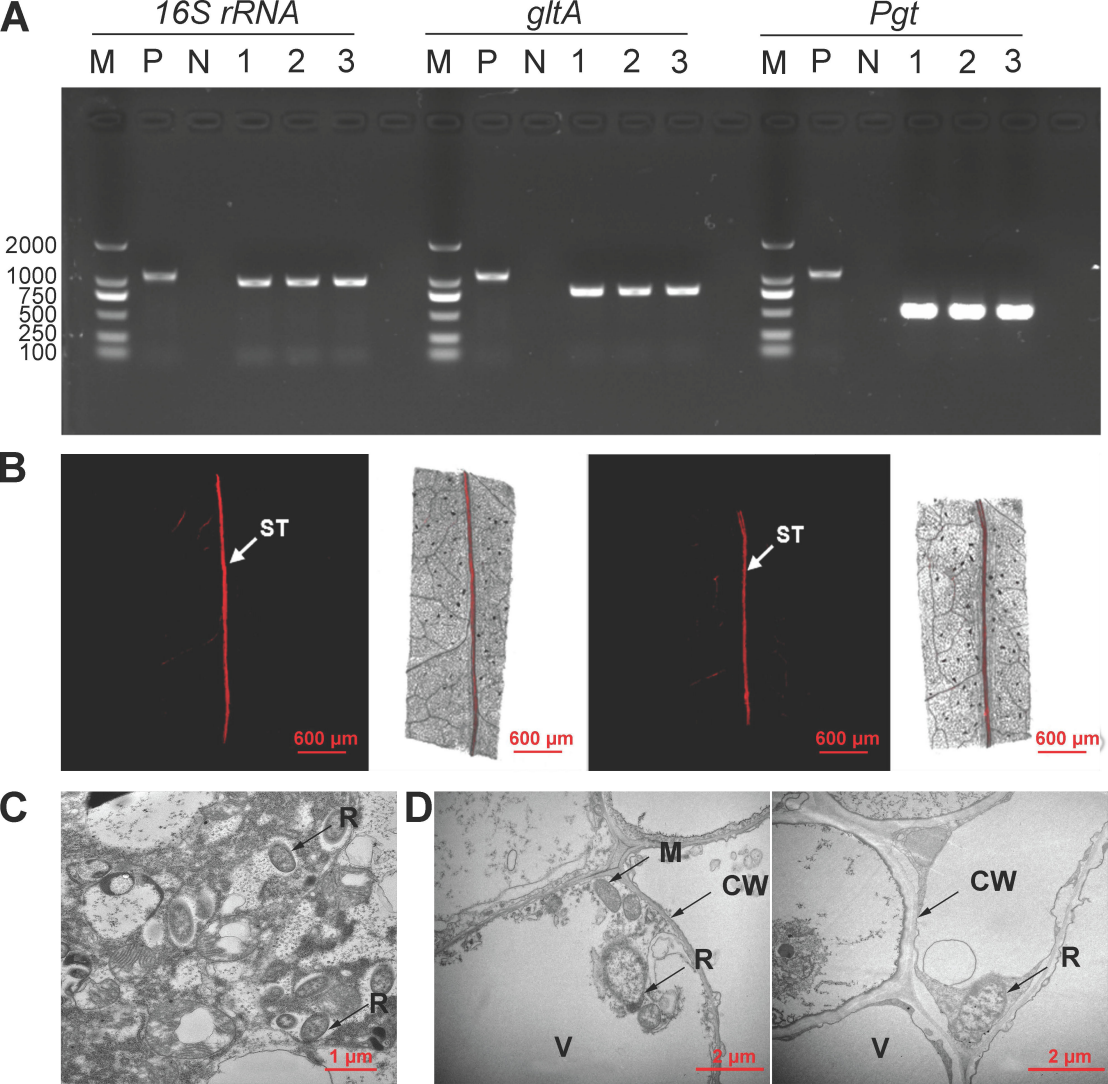
Location of *Rickettsia* in *Bemisia tabaci* and in tomato leaves. (**A**) PCR detection of *Rickettsia* in different tomato leaves. M: DNA marker; P: *16S rRNA* of *Portiera*, positive control; N: ddH2O, negative control. Lanes 1–3: DNA samples extracted from tomato leaf infested by *R*+ whiteflies, and the leaves situated above and below of an infested leaf, respectively. (**B**) *Rickettsia* visualization in tomato leaves with fluorescence *in situ* hybridization. Tomato leaves were taken from plants infested by *R*+ whiteflies. Left: sample of a leaf above the infested leaf; Right: sample of a leaf below the infested leaf. *Rickettsia*-specific *16S rRNA* was used as probe. (**C**) Detection of *Rickettsia* in the abdomen of an adult *R*+ whiteflies by transmission electron microscope; (**D**) Localization of *Rickettsia* in phloem sieve tube cells of a tomato leaf infested by *R*+ whiteflies. ST, phloem sieve tube; CW, cell wall of the plant phloem; V, vacuole; M, mitochondrion; R, *Rickettsia* endosymbiont.

The distribution and persistence of *Rickettsia* in tomato plants was detected by PCR, fluorescence *in situ* hybridization (FISH), and transmission electron microscopy (TEM). The titer of *Rickettsia* in tomato plants at different time periods was detected by quantitative real-time PCR (qRT-PCR). The PCR analyses confirmed that the endosymbiont, *Rickettsia belli*, can be transmitted from *B. tabaci* whiteflies into the phloem of tomato leaves. We also detected *Rickettsia* gene PCR products in the leaves adjacent to whitefly-infested leaves (Fig. 1A). The BLAST results of three genes specific for *Rickettsia*, namely, *gltA*, *Pgt,* and *16S rRNA*, confirmed that the *Rickettsia* endosymbionts from *B. tabaci* and tomato leaf samples were identical to each other. They fully matched the *gltA*, *Pgt,* and *16S rRNA* gene sequences of *Rickettsia* in GenBank (KX645660-KX645662).

The visualization using FISH confirmed that, after entering the tomato plant, *Rickettsia* was located exclusively inside the phloem vessels of plant leaves. From there, *Rickettsia* moved from the whitefly-infested leaf through the phloem to the younger and older neighboring leaves (Fig. 1B). There was no FISH signal found in the control plant leaves (Fig. S2).

The TEM images revealed the presence of *Rickettsia* in the *B. tabaci* adult abdomen (Fig. 1C). The *Rickettsia* cells are rod-shaped with a cell wall structure and are approximately 2.0 µm long and 0.5 µm wide. In tomato leaves, we found *Rickettsia* in the phloem sieve vessels. The *Rickettsia* cells in the tomato leaves were morphologically similar to those in the abdomen of *B. tabaci* adults though the size varied among individual cells (Fig. 1D).

**Fig 2:**
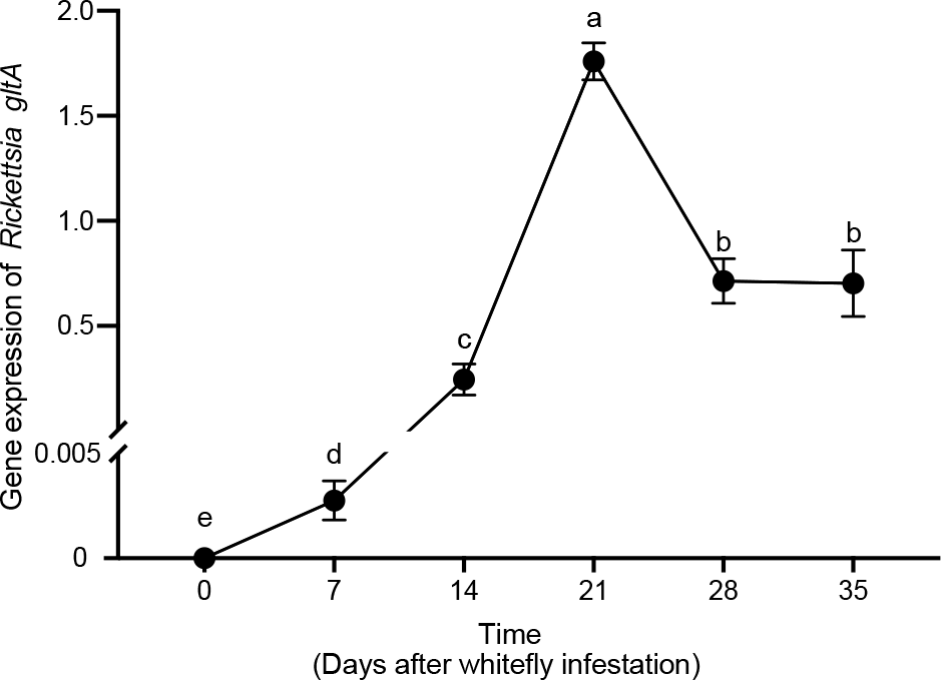
The persistence of *Rickettsia* in tomato leaves adjacent to a whitefly infested leaf. For *Rickettsia* detection, *glt A* qRT-PCR primers were used. The expression levels of two tomato housekeeping genes, *RuBisCo* and *β-actin*, were used for normalization. Different letters over the bars indicate statistically significant differences, error bars are standard deviations (*n* = 15; Duncan’s multiple range test, alpha = 0.05 level).

Once *Rickettsia* was transmitted to the tomato plant by the whitefly, they spread to undamaged leaves. The relative quantities of *Rickettsia,* as determined with qPCR, in the leaves neighboring the infested leaf increased during the first 21 days. After that, the quantities started to decrease (Fig. 2). The relative quantity of *Rickettsia* on the 21st day was approximately 300 times higher than that on the 7th day, and the relative quantities on the 14th, 28th, and 35th days were about 48, 175, and 132 times higher than that of the 7th day, respectively.

### Effects of *Rickettsia* infection on plant defense responses

The transcriptomics and defense response of tomato plants after *Rickettsia* entry were analyzed. Three cDNA libraries were sequenced: (1) tomato plants pre-infested with *Rickettsia* positive (*R+*) whitefly, (2) tomato plants pre-infested with *Rickettsia* negative (*R*−) whitefly, and (3) undamaged tomato plants (Ctrl). In our RNA-seq results, we found 7,157 (4,047 upregulated and 3110 downregulated); 6,538 (3,360 upregulated and 3,178 downregulated); and 2,583 (1,029 upregulated and 1,554 downregulated) differentially expressed genes (DEGs) in Ctrl vs *R*−, Ctrl vs *R+*, and *R*− vs *R+*, respectively (Tables S3 and S4; Fig. S3). A Venn diagram showed that 962 DEGs were commonly expressed among all the treatments (Fig. S4).

In the gene ontology (GO) analysis, we found 17 GO terms, 3 in biological process (GOBP), 7 in cellular component (GOCC), and 7 in molecular function (GOMF), were significantly enriched in both Ctrl vs *R−* and Ctrl vs *R+* (Fig. S5). However, these 17 GO terms were not significantly enriched in *R*+ vs *R−*. Between Ctrl vs *R*+ and *R*+ vs *R−*, 6 GOBP and 7 GOMF were significantly enriched in both comparisons. The Kyoto Encyclopedia of Genes and Genomes (KEGG) signaling pathway analyses revealed that plant-pathogen interaction (ko04626) and plant hormone signal transduction (ko04075) were significantly enriched (Fig. S6).

**Fig 3:**
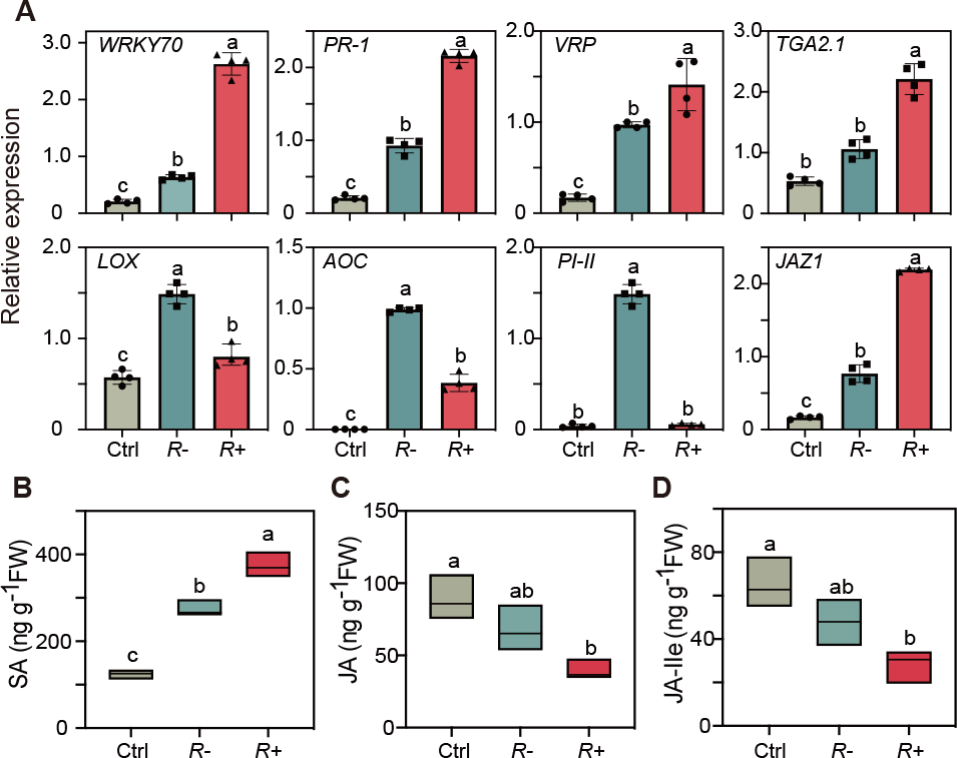
Effects of *Rickettsia* infection on defense responses in tomato. (**A**) Mean transcript levels (±SE, *n* = 3) of SA-(*WRKY70*, *PR-1*, *VRP*, and *TGA2.1*) and JA-regulated (*LOX*, *AOC*, *PI-II*, and *JAZ1*) genes in the undamaged plants (Ctrl), plants infested with *R−* whiteflies, or *R*+ whiteflies. The tomato household *RuBisCo* gene was used for normalization of the expression levels. The mean levels (±SE, *n* = 5) of SA (**B**), JA (**C**), and jasmonoyl-isoleucine (JA-Ile) (**D**) in the undamaged plants (Ctrl), plants infested with *R−* whiteflies (*R−*), or *R*+ whiteflies (*R+*) were measured. Different letters over the bars indicate statistically significant differences (Duncan’s multiple range test, alpha = 0.05 level).

To validate the RNA-Seq results, 16 DEGs with different expression patterns were selected for qPCR validation (Fig. S7). Similar results were obtained from both qPCR and RNA-Seq analyses: feeding by *R*+ whiteflies increased expression levels of SA-responsive *WRKY70*, *PR1*, *VRP*, and *TGA 2.1* compared with feeding by *R−* whiteflies (Fig. 3A). In contrast, expression levels of JA-responsive *PI-II*, *AOC,* and *LOX* genes were lower, while expression level of *JAZ1* gene was higher*,* in tomato plants fed upon by *R*+ whiteflies compared to the plants fed upon by *R*− whiteflies (Fig. 3A).

To further examine the effect of *Rickettsia* on tomato defenses, we measured the levels of endogenous salicylic acid (SA), jasmonic acid (JA), and JA-Ile in plants infested by *R*+ or *R−* whiteflies and undamaged plants. After 7 days infestation, the level of SA in plants infested by *R*+ whiteflies was significantly higher than that in plants infested by *R−* whiteflies (*P* = 0.02; Fig. 3B). In contrast, the levels of JA and JA-Ile in plants infested by *R*+ whiteflies were significantly lower than those in plants infested by *R−* whiteflies (JA: *P* = 0.01; JA-Ile: *P* = 0.02; Fig. 3C and D).

**Fig 4:**
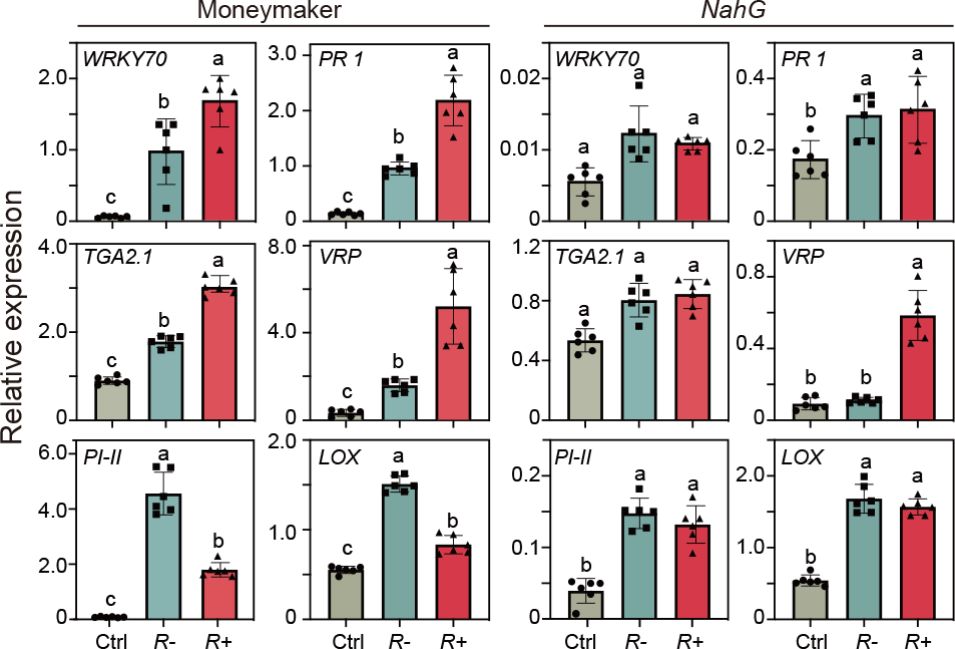
Transcript levels of SA- and JA-regulated genes in wild-type Moneymaker and SA-deficient NahG plants infested with *R−* whiteflies or *R*+ whiteflies. Values are untransformed means ± SE (*n* = 3). The tomato household *RuBisCo* gene was used for normalization of the expression levels. Different letters over the bars indicate statistically significant differences (Duncan’s multiple range test, alpha = 0.05 level). Ctrl, undamaged plants; *R−*, plants infested with *R−* whiteflies; *R+*, plants infested with *R*+ whiteflies.

To examine whether the increased SA levels affect the suppression of JA-regulated defense in infested *R*+ plants, we measured the expression of SA- and JA-regulated genes in Moneymaker (wild type) and SA-deficient *NahG* tomato plants infested by *R−* or *R*+ whiteflies. In Moneymaker, compared to feeding by *R−* whiteflies, feeding by *R*+ whiteflies increased the expression of SA-regulated *PR-1*, *TGA2.1*, and *VRP* but decreased the expression of JA-regulated *PI-II* and *LOX* (Fig. 4). In contrast, the suppression of SA-regulated genes (*PI-II* and *LOX*) and the induction of JA-regulated genes did not occur in *NahG* plants infested by *R*+ whiteflies (Fig. 4). These results indicate that negative cross-talk between the JA and SA pathways was involved in JA defense suppression by *Rickettsia* infection. The expression patterns of *VRP* were similar on both plant types, implying that its expression is independent of SA.

### *Rickettsia* infection promotes the susceptibility of tomato plants to herbivores

**Fig 5:**
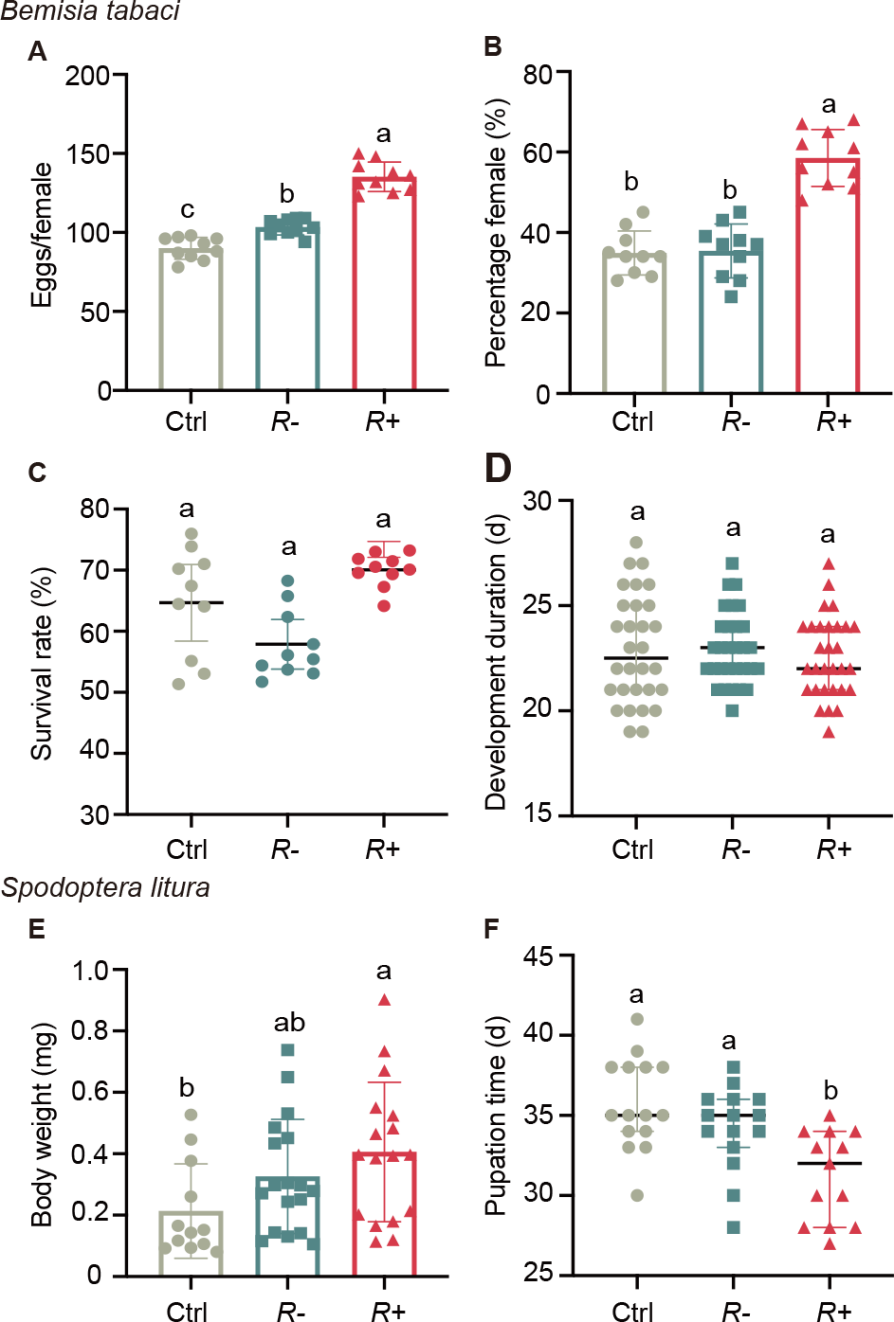
Infection of *Rickettsia* increases susceptibility to insect herbivores. (**A**) Average fecundity, (**B**) sex ratio of the progeny, (**C**) survival rate from egg to adult, (**D**) developmental period from egg to adult of *Bemisia tabaci* infested on different tomato plants, (**E**) larval mass at day 22, and (**F**) pupation time of *S. litura* fed on different tomato plants. Values are means ± SE (*n* = 10). Different letters over bars indicate significant differences between treatments (Duncan’s multiple range test, alpha = 0.05 level). Ctrl, undamaged plants; *R−*, plants pre-infested by *R−* whiteflies; *R+*, plants pre-infested by *R*+ whiteflies.

To investigate whether *Rickettsia* infection affects the susceptibility of tomato plants to subsequent infestations by herbivores, we evaluated the performance of whitefly and *S. litura* larvae on plants pre-infested by *R−* or *R*+ whiteflies. Female whiteflies laid more eggs on plants pre-infested by *R*+ whiteflies than on plants pre-infested by *R*− whiteflies (Fig. 5A). In addition, the female proportion of progeny reared on plants pre-infested by *R*+ whiteflies was about 50% higher than on plants pre-infested by *R*− whiteflies (Fig. 5B). Yet, *Rickettsia* infection did not significantly affect the survival rate and developmental time of whitefly (Fig. 5C and D).

Feeding *S. litura* larvae on plants that were pre-infested with *R*+ whiteflies resulted in larvae with higher body weights and shortened pupation times as compared to feeding them on control plants (Fig. 5E and F). In contrast, the body weight and pupation time of *S. litura* reared on plants pre-infested with *R−* whiteflies showed no such effect (Fig. 5E and F). These results indicate that *Rickettsia* infection played a crucial role in suppressing the defense of tomato against herbivores.

### *Rickettsia* infection induced resistance of tomato plants against pathogens

**Fig 6:**
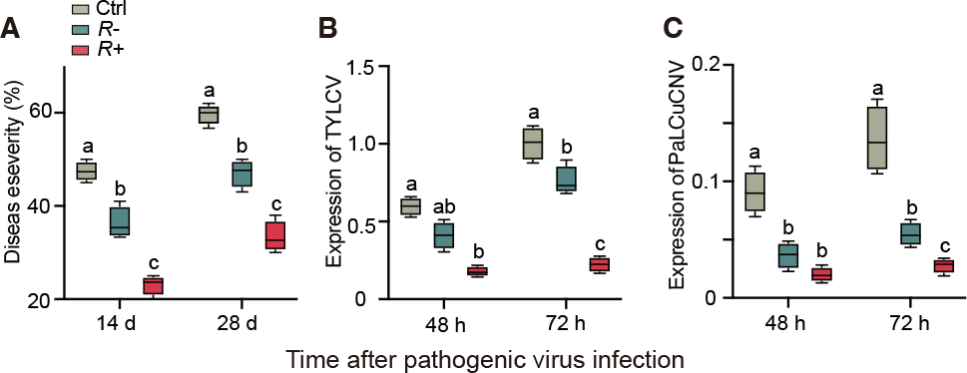
Infection of *Rickettsia* induces resistance against pathogens. (**A**) Disease severity assessment of *V. dahliae*, (**B**) expression of *TYLCV*, and (**C**) expression of *PaLCuCNV* on different tomato plants. The tomato household *RuBisCo* gene was used for normalization of the expression levels. Values are means ± SE (*n* = 3). Different letters over bars indicate significant differences between treatments (Duncan’s multiple range test, alpha = 0.05 level). Ctrl, undamaged plants; *R−*, plants pre-infested by *R−* whiteflies; *R+*, plants pre-infested by *R*+ whiteflies.

**Fig 7:**
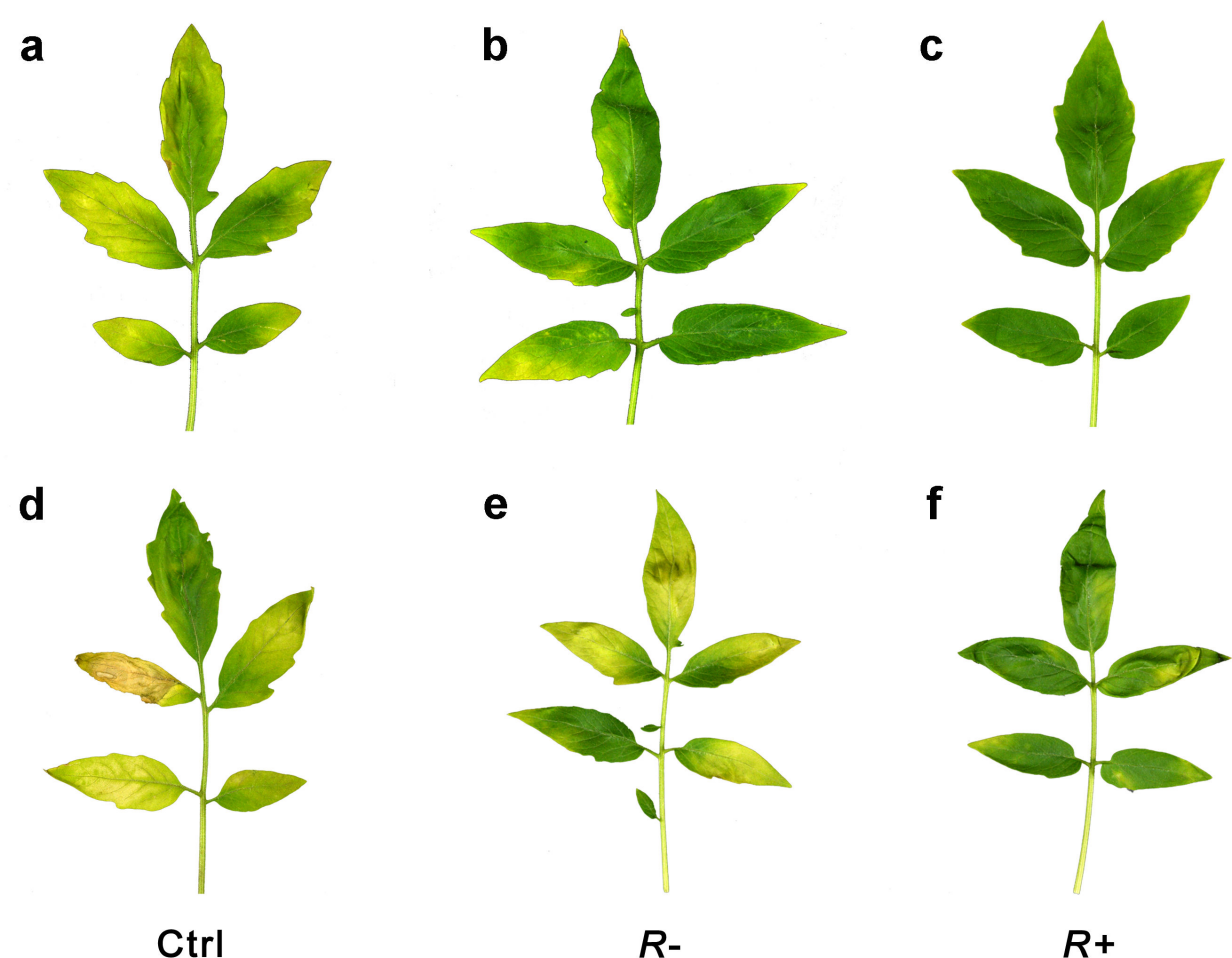
The disease symptoms of *V. dahliae* infesting different tomato plants. (a–c) *V. dahliae* symptoms of Ctrl, *R−* and *R*+ plants at day 14, (d–f) *V. dahliae* symptoms of Ctrl, *R−* and *R*+ plants at day 28. Treatment *R+*: tomato pretreated with *R+ B. tabaci* for 7 days, *R−*: tomato pretreated with *R− B. tabaci* for 7 days, Ctrl: uninfested tomato leaves as controls.

To investigate if the *Rickettsia* infection affected the resistance of tomato plants against pathogens, we measured the performance of the fungal pathogen *V. dahlia* and the expression levels of the viruses TYLCV and PaLCuCNV, which are all known to be vectored by whiteflies. At the 14 and 28 days after *V. dahliae* infection, the wilt symptoms caused by *V. dahliae* on plants pre-infested with *R*+ whiteflies were significantly lower than those on plants pre-infested with *R−* whiteflies (Fig. 6A and 7). The feeding by *R*+ whiteflies also suppressed expression levels of TYLCV and PaLCuCNV at 72 h as compared to those by *R*− whiteflies but not at 48 h after virus infection (Fig. 6B and C). These results imply that the infection with *Rickettsia* positively increased the resistance of tomato plants against pathogens.

## DISCUSSION

Manipulation of the plant immune system by insects has largely focused on effectors that are produced by the plant antagonists themselves (29–33), or microorganisms that they carry with them (34). Here, we revealed the mechanism how the transmission of an endosymbiont by an insect to a host plant enhances the suitability of the plant for the insect, which was also previously reported by Chung et al. (34) and Chen et al. (35). Various studies have revealed that host plants can vector the horizontal transmission of endosymbionts between different populations, or even different species, of phloem feeding insects (20, 21, 23, 24, 36). On the other hand, herbivore-associated microorganisms can be transmitted to a plant and spread throughout a plant and persist there while the herbivore insect continuously feeds (23, 37). Here, we reveal that *Rickettsia* is, indeed, vectored by whiteflies and infects tomato plants (Fig. 1). It spreads and stays active in the phloem for at least 5 weeks (Fig. 2). Gene-expression and phytohormone analyses further showed that the infection of *Rickettsia* vectored by whiteflies enhances SA-regulated defenses, but suppresses JA-regulated defenses (Fig. 3), which are defenses that strongly affect whitefly performance (38–40). Our study demonstrates that endosymbionts can infect and modify plant defenses in a manner that benefits their insect host.

Various studies have shown that microbial symbionts can provide herbivore insects with essential nutrients including B vitamins and also enhance their defenses against predators (15, 41), but the role of insect symbionts as effectors in modifying plant-insect interactions has received scant attention. In apple, *Wolbachia*-infected leaf miners (*Phyllonorycter blancardella*) elicit a green-island phenotype, which preserves photosynthetically active tissue in senescent leaves and indirectly enhances leaf miner performance (42). In maize, it has been proposed that *Wolbachia* may suppress plant defenses against the western corn rootworm (*Diabrotica virgifera virgifera*) (43), but this has been questioned (44). It remains uncertain that insects secrete symbionts or symbiont-derived compounds into plants during feeding for the purpose of plant manipulation. Our study reveals that *Rickettsia* associated with whiteflies are secreted into tomato leaves and stay active and reproduce for more than 1 month. Moreover, the plant defense suppression by *Rickettsia* was found to enhance the performance of phloem-feeding *B. tabaci* and chewing *S. litura* caterpillars (Fig. 5). The long-term colonization of *Rickettsia* in a tomato plant in our study has shown that it can change the plant defenses. However, previous studies revealed that some aphid symbiotic bacteria, which are transmitted into the plant by aphids, can manipulate the plant defenses via attenuating the plant’s volatile emissions although these bacteria do not multiple themselves within the plant (45). This kind of manipulation may occur through the symbiont effectors delivered with aphid saliva, or through the deposition of symbionts on the plant surface via honeydew that are then injected into the plant while aphids are piercing (46, 47). In our current study, the same situation may also exist; therefore, further studies need to be undertaken to isolate the manipulators of plant defenses including the symbiotic bacteria in plant phloem and in the honeydew of phloem-sucking insects, as well as in the saliva of piercing vector insects. All these findings imply that insect symbionts play a more intricate role in the interaction between plants and insects than previously thought (15).

Our validation experiments using SA-deficient *NahG* mutants and wild-type tomato plants revealed the importance of crosstalk between the SA and JA signaling pathways. *Rickettsia*-mediated defense suppression was not observed in SA-deficient *NahG* plants, indicating that suppression of JA-regulated defenses is linked to the upregulation of the SA signaling pathway (48). Indeed, it has been well documented that pathogens may manipulate the antagonistic effects between JA and SA and, thereby, suppress host plant defenses so enhancing the performance of insect vectors (49, 50). It is important to note that also in *NahG* plants, feeding by *R*+ whiteflies increased the expression level of the pathogen-resistance gene *VRP* compared to feeding by *R*− whiteflies (Fig. 4). This indicates that *Rickettsia* presence enhances the expression of *VRP* via a mechanism that is not dependent on the SA or JA pathway.

The behavioral and performance assays showed that *R+ B. tabaci* females laid more eggs than *R− B. tabaci* females (Fig. 5A). There may be three non-exclusive reasons for this. First, carrying *Rickettsia* might increase whitefly fecundity as we also found on cotton plants (Shi et al., unpublished data). Second, by suppressing the JA-mediated plant defenses, the *Rickettsia* infection may increase plant quality and, thus, indirectly the fitness of *B. tabaci*. Third, the females may detect the superior quality of the *Rickettsia*-infected plants and prefer to lay more eggs on them than on uninfected plants. The females did not only lay more eggs on *R*+ plants, but these eggs also resulted in more female progeny, which may further increase their fitness (Fig. 5B).

It is important to note that feeding by *R*− whiteflies triggered the expression of JA-regulated defense genes (*LOX*, *AOC*, and *PI-II*; Fig. 3A). This means that, without a *Rickettsia* infection resulting from whitefly feeding, host plants can perceive the infesting insects and respond with appropriate defense responses. A similar phenomenon was observed in the interaction between the Colorado potato beetle (*Leptinotarsa decemlineata*) and tomato plants, whereby larvae without symbionts activated stronger JA-regulated defenses than those with symbionts, which resulted in reduced larval growth (36). In the current study, the activation of JA-regulated genes induced by *R−* whiteflies seems not to significantly affect the survival and development of whitefly adults (Fig. 5C and D) as previously found (51), but compared to infections by *R*+ whiteflies, it reduces fecundity (Fig. 5A) and affects the insect’s sex ration (Fig. 5B).

Our study revealed that whitefly-infestation significantly reduced the plants’ susceptibility to *Verticillium* wilt (Fig. 7) and to the whitefly-vectored viruses TYLCV and PaLCuCNV (Fig. 6). We deduce that by itself whitefly induction of the SA-pathway enhances the resistance to these important plant pathogens and that *Rickettsia* further synergizes the plant responses (Fig. 3 and 4), making the plants even more resistant (Fig. 6). As far as we are aware, this is the first study to show that feeding by whitefly induces resistance to pathogen infestation and that *Rickettsia* further synergizes this effect.

In conclusion, our study answers two important and unresolved questions in whitefly-plant interactions. Walling (52) hypothesized that the mechanism by which the whitefly *B. tabaci* down-regulates JA defenses via SA cross-talk in host plants involves a salivary component synthesized by the whitefly or one of its endosymbionts. In another insightful review on the horizontal transmission of endosymbionts via the host plants, Chrostek et al*.* (19) asked the question “how do insects symbionts influence plants?” after their horizontal transmission. Here, we show that the horizontal transmission of *Rickettsia* from whitefly to plants enhances the plants’ suitability to insect herbivores and simultaneously makes them more resistant to pathogens. In addition, a field survey from 2011 to 2022 showed that the *B. tabaci* population that harbors *Rickettsia* was gradually increasing and becoming the dominant species in Guangdong, China (Qiu et al., unpublished data). These data further indicate that the bacterial symbionts may be consequently changing the structure of the herbivore via the plant defenses at a community level. As far as we know, our study represents the first report of an adaptive transmission by insects of an endosymbiont to plants in order to manipulate the plants’ immunity in a manner that benefits the insect as well as the symbiont. If and how the endosymbiont may also provide certain benefits to plants is worthy of further investigation.

## MATERIALS AND METHODS

### Host plants

Seeds of the tomato plant *Lycopersicon esculentum* Miller (var. Xinjinfeng no. 1, Changhe Seed Co. Ltd., Guangzhou), a salicylic acid-deficient *NahG* mutant, and its wild type (variety Moneymaker) were sown in 15 cm diameter plastic pots containing a soil-sand mixture (10% sand, 5% clay, and 85% peat). The seedlings were cultured at ambient temperature and photoperiod in a glasshouse at South China Agricultural University (SCAU) (with a mean of 27.5°C and 12.2 h light per day during September-October, 2017). The plants were watered as required and were used for experiments at the six to eight expanded leaf stage. All the leaf surfaces were disinfected with 75% alcohol and allowed to air dry to remove other phyllospheric microorganisms before undertaking experiments.

### Insects and pathogens

#### *Phloem feeding whitefly* Bemisia tabaci

We screened two populations of *B. tabaci* belonging to the Middle East-Asia Minor 1 cryptic species (MEAM1, formerly B biotype) for the current study. One population is *Rickettsia* positive (*R+*), and the other is *Rickettsia* negative (*R*−). Both populations share the same genetic background (Supplementary Material). The *R*+ and *R− B. tabaci* populations were mass reared on cotton plants (*Gossypium hirsutum* L. var. Lumianyan no. 32) before experimental use. Whiteflies were reared in climate-controlled chambers (26 ± 1°C, RH 75 ± 10% L:D = 14:10; RXZ 500, Jiangnan Instrument Co. Ltd., Ningbo, China).

#### *Chewing herbivore* Spodoptera litura

*Spodoptera litura* larvae (*Rickettsia* negative [see the supplemental material]) were originally collected on broccoli plants at the training farm of SCAU, Guangzhou in the fall of 2015. Thereafter, it was mass reared on artificial diet (53) for at least eight generations. Both the whitefly and the noctuid insects were reared under the same conditions as described in the above section.

#### *Pathogenic fungus* Verticillium dahliae

*V. dahliae* (strain v991) was initially grown on potato dextrose agar (PDA) media and stored at 4°C before use (see the supplemental material).

#### Viruses

Clones of tomato yellow leaf curl virus (TYLCV; GenBank accession no. AM282874) and Papaya leaf curl China virus (PaLCuCNV; GenBank accession no. AM691554) were used for virus inoculation and transmission.

### *Rickettsia* transmission, distribution, and persistence

#### *Horizontal transmission of* Rickettsia *from whitefly to tomato plants*

To transmit *Rickettsia* to the tomato plants, 30 pairs of 24–48 h old *R+ B. tabac*i adults were collected from the *R*+ subcolony and released into a nylon bag (10 × 15 cm, 70 mesh/cm^2^) that covered one tomato composite leaf. After 1 week of whitefly feeding, the distribution and persistence of *Rickettsia* in the tomato plant was examined as described below.

#### *The distribution and persistence of* Rickettsia *in tomato plants*

The persistence and localization of *Rickettsia* in the tomato plants was assessed using PCR, fluorescence *in situ* hybridization (FISH) and transmission electron microscopy (TEM) techniques.

##### PCR detection

One upper or lower tomato leaf neighboring the leaf infested with *R+* whitefly (about 2–3 cm distance between each of the two leaves on the stem) was cut and homogenized in lysis buffer for DNA extraction (TIANamp Genomic DNA Kit, Tiangen Biotech Co. Ltd, Beijing, China). The specific primers used for *Rickettsia* detection were citrate synthase (*gltA*), phosphoglycerol transferase (*Pgt*), and *16S rRNA* genes, which were amplified according to Caspi-Fluger et al*.* (21) and Gottlieb et al*.* (54) (Table S1). *Portiera aleyrodidarum* DNA was used as a positive control, and ddH_2_O was used as a negative control. All the *gltA*, *Pgt,* and *16S rRNA* genes of *Rickettsia* amplified from plant leaves were sequenced and then searched using BLAST in GenBank to confirm their strains. Twelve tomato plants were used in the PCR detection.

##### *Fluorescence* in situ *hybridization detection*

A strip of tomato leaf (10 mm × 5 mm, 0.01 g) was cut longitudinally along both sides of the midrib from the uninfested leaf neighboring the leaf infested with *R*+ whitefly. This leaf strip was used to visually inspect for *Rickettsia* presence and localization using FISH. The leaves were placed in Carnoy’s fixative, and FISH was performed with the symbiont-specific *16S rRNA* probe for *Rickettsia* (Rb1-Cy5: 5′-TCCACGTCGCCGTCTTGC-3′) (54). Stained tomato leaves were mounted and viewed under a Nikon eclipse Ti-U inverted microscope. Healthy tomato leaves infested with *R−* whitefly and leaves infested with *R*+ whitefly but without symbiont-specific *16S rRNA* probe hybridization were used as negative controls. Eighteen tomato plants were used in the FISH detection.

##### Transmission electron microscope detection

The *Rickettsia* localization in tomato leaves was detected with a transmission electron microscope (TEM) according to the method of Li et al*.* (24). Briefly, samples of *Rickettsia* infested tomato leaves (1.0 mm × 0.5 mm) were fixed in 4% glutaraldehyde in cacodylate buffer (pH 7.4) at 4°C for 24 h and then overnight in 1% osmium tetroxide. The fixed leaf samples were dehydrated through an alcohol series and embedded in Spurr’s resin. Ultrathin sections were collected on copper grids with a single slot, stained with 1% uranyl acetate and lead citrate, and finally examined under transmission electron microscopy (JEOL, Tokyo, Japan). Nine tomato plants were used in the TEM detection.

##### Quantitative real-time PCR detection

The persistence of *Rickettsia* in tomato leaves was quantified with quantitative real-time PCR (qPCR) after 7, 14, 21, 28, and 35 days of *R*+ whitefly feeding (*n* = 3 per time point). The leaf sampling and DNA extraction were as described above. The primers used for *Rickettsia* qPCR detection were *gltA,* and two housekeeping genes of the tomato plant, *RuBisCo* and *β-actin*, were selected as internal controls for data normalization and quantification (38). Statistically significant differences of the relative quantity of *Rickettsia* were determined by analyzing the data with ANOVA followed by Duncan’s test at alpha = 0.05 (SPSS vs 18.0, SPSS Inc. Chicago, USA).

### Plant pre-treatments with whitefly

In order to determine the response of tomato plants to whitefly feeding and *Rickettsia* persistence, tomato plants were grown as described above for 4 weeks after which they were subjected to the following three treatments: (1) plants were pre-infested with *R*+ whiteflies for 7 days (*Rickettsia* positive [*R+*) (2); plants were pre-infested with *R*− whiteflies for 7 days (*Rickettsia* negative [*R−*]) (3); uninfested plants (as control plants in the experiment, hereafter abbreviated to “Ctrl”). Approximately 60 pairs of *R*+ or *R−* whitefly adults (24–48 h old) were collected and divided into two nylon bags that covered the upper and lower surfaces of one tomato leaf, whereas control plants received empty sleeves.

The *R*+ and *R− B. tabaci* adults were used to induce the plant defense with a 7 day-infestation in all experiments. This was based on our previous finding (55) that, when using an exotic biological or chemical factor to induce plant defenses, the endogenous JA and SA usually displayed distinct changes from the 4th day onwards and peaked on the 11th day after whitefly feeding. Thus, we chose the plants pre-infested with whiteflies for 7 days to investigate the JA- and SA-affected performance of the herbivores and pathogen in our current study.

### Transcriptomics analysis of tomato plants feeding with different whiteflies

The differentially expressed genes (DEGs) in different treatments of tomato plants were analyzed. The middle tomato leaves neighboring the whitefly-infested ones were harvested for RNA extraction after 7 days of whitefly feeding. Six biological replicates (leaves) in each treatment were randomly split into two groups of three plants each for RNA sequencing and qPCR analysis, respectively.

#### RNA sequencing and bioinformatics analysis

The total RNA of a tomato plant was extracted using TRIzol Reagent (Invitrogen, Guangzhou). The mRNA was purified using the NucleoTrap mRNA kit (Macherey-Nagel, Düren, Germany). Then, the cDNA samples were constructed using the Standard cDNA Synthesis Kit (Takara, Japan) according to Marioni et al*.* (56). All cDNA were sent to Beijing Genomics Institute (Shenzhen, China) for sequencing with the BGISEQ-500RS platform. SOAPnuke1.5.6 was used for reads trimming to remove adaptors and low-quality bases. HISAT (version 2.0.4) and Bowtie2 (version 2.2.5) were used to index reference genome and reads mapping. Tomato genome annotation ITAG3.2 was used as the reference genome. RSEM (version 1.2.8) was used to generate the count matrix. Reads count was normalized with FPKM, and NOISeq was used to identify differentially expressed genes (57). We selected the genes with a log_2_ fold change ≥1.5 and deviation probability value ≥0.8 of each plant sample for further analyses. Gene set enrichment analysis was performed with AgriGO (version 2.0) (58). All three Gene Ontology (GO) (i.e., Molecular Function, Biological Process, and Cellular Component) and KEGG Plant pathways (59) were used. An adjusted *P* value < 0.05 cutoff was used for selecting significantly enriched GO terms and KEGG pathways.

#### Validation of differential expression genes by qPCR

To validate the RNA seq result, 16 DEGs were randomly selected for further qPCR validations (Fig. S2). To identify potential genes that were involved in the response of the tomato plant caused by exogenous *Rickettsia*, we validated the expression changes of 6 DEGs associated with immune response-related GO terms and KEGG pathways. These genes are *WRKY70* and *PR1-(P4)* (pathogenesis-related protein 1), indicative for activation of the salicylic acid (SA) pathway, and *AOC* (allene oxide cyclase), *LOX* (linoleate 13S-lipoxygenase 2-1), *PI-II* (proteinase inhibitor II), and *JAZ1* (jasmonate ZIM-domain protein 1), indicative of a response in the jasmonic acid (JA) pathway. In addition, we selected two pathogen-resistance genes, *VRP* (*Verticillium* wilt disease resistance 2) and *TGA2.1*, for further quantification by qPCR.

The primers and protocol for qPCR quantification are specified in Table S2. The PCRs were performed on Bio-Rad CFX96 Real-Time System using SYBR Green (Bio-Rad, USA). The relative expression levels were calculated with the 2^−ΔΔ*CT*^ method and further log_2_ transformed, and the *RuBisCo* gene (ribulose-bisphosphate carboxylase) of the tomato plant was used as a reference gene.

### Phytohormone analysis of tomato plants feeding with different whiteflies

#### Analysis of JA, JA-Ile and SA

The phytohormones JA, JA-Ile, and SA were analyzed as described by Engelberth et al*.* (60) with modification. In brief, plant material (250–300 mg) was frozen and ground in liquid nitrogen. For quantification purposes (9, 10), dihydro-JA (15 ng; Sigma-Aldrich, St Louis, MO, USA) and D6-SA (20 ng; CDN Isotopes, Pointe-Claire, Quebec, Canada) were added as internal standards with 2 mL of 80% methanol. JA, JA-Ile, SA, and the internal standards were partitioned to an aqueous phase by centrifugation and vaporization. Subsequently, they were extracted from the aqueous phase with an equal volume of ethyl acetate and then dried. The dried extract was resuspended in 0.1 M acetic acid and loaded onto a C18 column (Waters Company, Milford, MA, USA). The C18 column was sequentially eluted with a series of solvent mixtures [acetic acid/methanol (vol/vol) at 83/17, 60/40, and 40/60]. The effluents of the last 4 mL in 40% methanol and the first 3 mL in 60% methanol were collected. After evaporation of the solvent and esterification of the residue using excess ethereal diazomethane, samples were analyzed using a gas chromatograph coupled to a mass selective detector (6890N/5973 MSD, Agilent Technologies, Inc., Palo Alto, CA, USA), which was operated in electron impact ionization mode. The compounds in the samples were separated on an HP-5-MS column (30 mm × 0.25 mm × 0.25 mm; 19,091 S-433, J&W Scientific, Agilent Technologies). JA, JA-Ile, and SA were quantified by correlating the peak area (extracted ion) of the compound with the peak area of the respective deuterated internal standard. Instead of measuring D6-MeSA, we measured D4-MeSA due to the loss of two deuterium ions during sample preparation.

#### Validation of related expression genes in SA-deficient NahG plants

Based on the results of the DEGs and qPCR analyses, the role of SA and cross-talk between the JA and SA signal transduction pathways were further studied. To do so, the same treatments (*R+*, *R−*, and Ctrl, *n* = 6 per treatment) were applied to SA-deficient *NahG* mutant tomato plants and their wild-type, cv. Moneymaker. The expression of *WRKY70*, *PR1*, *AOC*, *LOX*, *PI-II*, *VRP*, and *TGA2.1* was quantified by qPCR as described above. Three technical qPCR replicates were analyzed for each biological replicate, and the statistically significant differences between the relative expression quantities of DEGs were analyzed using ANOVA followed by Duncan’s test.

### Effects of *Rickettsia* persistence on the performance of *Bemisia tabaci*

Tomato plants in this experiment were pre-treated as above. Approximately 30 pairs of *R+ or R− B. tabaci* adults were allowed to feed for 7 days before being removed. Hereafter, one pair of *B. tabaci* adults (24–48 h old) was introduced into another nylon bag (20 × 30 cm) that covered one healthy, fully expanded tomato leaf of a pre-treated plant. The fecundity of the female (numbers of eggs/female) was examined until the female died. In a second experiment, 30 pairs of *B. tabaci* adults (24–48 h old) were introduced into another nylon bag covering one healthy, fully expanded leaf of a pre-treated plant. The adult whiteflies were removed after 24 h. Then, the development of whitefly progeny including the survival (egg-adult), developmental time of whitefly nymphs as well as the sex ratio of F1 generation adults were assessed.

There were three biological repeats for each treatment, and each repeat included 10 plants. Statistically significant differences in fecundity, survival, development time of *B. tabaci* among the differently treated tomato plants were analyzed using ANOVA followed by Duncan’s test.

### Effects of *Rickettsia* persistence on the performance of *Spodoptera litura*

Tomato plants in this experiment were pre-treated as above. After 7 days of *R*+ or *R*− whitefly feeding, one freshly molted 2nd instar *S. litura* larva was introduced onto an uninfested leaf of *R+*, *R-*, and Ctrl plants within a nylon bag using a soft brush. On the 22nd day, the body mass of the *S. litura* larva was measured with a digital balance (0.1 mg precision, CP214, Ohaus Co. Ltd., China). In addition, the period of *S. litura* from larvae to pupae was recorded. The experiments were repeated three times, and each time 10 individuals of *S. litura* larvae were investigated for every treatment. Statistically significant differences in the developmental time and body weight of *S. litura* fed on the differently treated plants were analyzed using ANOVA followed by Duncan’s test.

### Effects of *Rickettsia* persistence on the performance of a pathogenic fungus

Before plant infection, the conidia of *V. dahliae* were plated on a PDA medium followed by 10-day incubation at 26°C, after which the conidial suspension was filtered through two layers of Miracloth (Calbiochem, USA) and then pelleted by low speed (800 × *g*) centrifugation. Finally, a suspension of 1 × 10^7^ conidia/mL was prepared for plant infection. Tomato plants for experiments were pre-infested with whitefly as above to yield *R+*, *R−,* and Ctrl plants. After 7 days of feeding by *R*+ or *R−* whitefly, we injected 1.0 mL conidia suspension of *V. dahliae* into the roots of the differently treated tomato plants (at the soil surface) using a micro-syringe. Then, the infection symptoms of *V. dahliae* on *R+*, *R*−, and Ctrl plants were observed every 7 days. The disease symptoms caused by *V. dahliae* were classified following the method of Markakis et al*.* (61) (Supplementary Material). Experiments were repeated 3 times, each time with 10 replicates (plants) per treatment. Statistically significant differences in the disease indexes among differently treated plants were analyzed using ANOVA followed by Duncan’s test.

### Effects of *Rickettsia* persistence on the performance of pathogenic viruses

Fifty viruliferous whiteflies collected from TYLCV- or PaLCuCNV-infected tomato plants were introduced into a ventilated cage (21.0 cm high, 13.5 cm width) that contained one *R+*, *R−*, or Ctrl plant. After 48 or 72 h of infestation by viruliferous whiteflies, leaf tissues collected from three plants were pooled as one sample. Leaf samples were directly frozen in liquid nitrogen and stored at −80°C for subsequent gene-expression analysis. The gene expression of *TYLCV* and *PaLCuCNV* was quantified by qPCR as described above. The *RuBisCo* gene was used as endogenous control gene. Primers used for quantitative RT-qPCR are given in Table S2. Three technical qPCR replicates were analyzed for each biological replicate, and the gene expression data of *TYLCV* and *PaLCuCNV* comparing Ctrl plants and virus-infected plants were analyzed using ANOVA followed by Duncan’s test. Each experiment was repeated with three biological replicates.

## Data Availability

The *gltA*, *Pgt*, and *16S rRNA* gene sequences of *Rickettsia* endosymbionts in whitefly and tomato plants were deposited in GenBank with accession numbers of KX645660-KX645662.

## SUPPLEMENTAL MATERIAL

Supplemental file mbio.02448-23-s0001.docx Supplemental text, tables, and figures.

ASM does not own the copyrights to Supplemental Material that may be linked to, or accessed through, an article. The authors have granted ASM a non-exclusive, world-wide license to publish the Supplemental Material files. Please contact the corresponding author directly for reuse.
