# Supplementary material for: Rickettsia transmission from whitefly to plants benefits herbivore insects but is detrimental to fungal and viral pathogens
Source: mBio. 2024 Feb 5;15(3):e02448-23. doi: 10.1128/mbio.02448-23 (PMC10936170; doi:10.1128/mbio.02448-23)
Supplement: Supplemental file — Supplemental text, tables, and figures. [file mbio.02448-23-s0001.docx]

***Rickettsia* transmission from whitefly to plants benefits herbivore insects but is detrimental to fungal and viral pathogens**

Pei-Qiong Shi^1^, Lei Wang^1^, Xin-Yi Chen^1^, Kai Wang^2^, Qing-Jun Wu^3^, Ted CJ Turlings^4*^, Peng-Jun Zhang^5*^, Bao-Li Qiu^1*^

^1^ Engineering Research Center of Biotechnology for Active Substances, Ministry of Education, Chongqing Normal University, Chongqing 401331, China

^2^ Department of Computational Medicine and Bioinformatics, School of Medicine, University of Michigan, Ann Arbor, MI48197, United States

^3^ Institute of Vegetables & Flowers, Chinese Academy of Agricultural Sciences, Beijing 100081, China

^4^ FARCE Laboratory, Institute of Biology, University of Neuchâtel, CH-2000 Neuchâtel, Switzerland

^5^ College of Life and Environmental Sciences, Hangzhou Normal University, Hangzhou 311121, China

***Corresponding author:**

Bao-Li Qiu, PhD, Professor,

E-mail: baoliqiu@cqnu.edu.cn

Peng-Jun Zhang, PhD, Professor,

E-mail: [Peng_junzhang@hotmail.com](mailto:Peng_junzhang@hotmail.com)

Ted CJ Turlings, PhD, Professor,

Email: [ted.turlings@unine.ch](mailto:ted.turlings@unine.ch)

**Supplementary Materials**

**The origin of *Bemisia tabaci Rickettsia* positive and negative populations**

Both the *Rickettsia* positive (*R*+) and negative (*R*-) populations originated from one ancestral *B. tabaci* MEAM1 population, which was collected from eggplant (*Solanum melongena*) grown at the Engineering Technology Research Center of Pest Biocontrol, South China Agricultural University (SCAU) in 2015. Following this, the population was reared on cotton plants under laboratory conditions (at 26±1 °C, RH 75±10% L:D=14:10) where the cryptic species was monitored monthly using the COI gene (Qiu et al., 2009). The infection rate of *Rickettsia* in this ancestral population was about 65% with little wave depending on the season and generations. The *R+* and *R-* sub-colonies were screened with the procedures of “single-pair method” that we previously established (Liu et al., 2020). Briefly, the original population of *B. tabaci* MEAM1 collected from field has a relatively low infection rate of *Rickettsia* (< 35%). When the *B. tabaci* adults newly emerged, unmated males and females were randomly selected, and released into a leaf cage that covered a healthy plant leaf in order to oviposit F1 generation eggs. Following 6 days of oviposition, the parents were recaptured and used for PCR detection. The F1 progeny, for which parents were either Rickettsia positive or negative, were used to produce the F2 generation, and similarly in turn for the F3, F4 and F5 generations respectively; if the infection status of *Rickettsia* was consistent in the F1 to F5 generations, then the populations can be used as *Rickettsia* positive or negative lines for further experiments.

With the screening of single-pair method, the *R+* and *R-* sub-colonies shared the same genetic background although they are *Rickettsia* infected or absent.

**The origin of *Verticillium* wilt pathogen**

The *Verticillium dahlia* strain v991 was provided by Dr. Wenwei Zhang (Institute of Plant Protection, Chinese Academy of Agricultural Sciences), which was originally isolated from infected cotton plants in fields of Beijing, and was cultured on PDA media.

**The origin of *Spodoptera litura***

Larvae *of S. litura* were reared with artificial diet (Gupta et al. 2005) in a climate chamber at 26±1 °C, RH 75±10% L:D=14:10.

**Supplementary Methods**

**1. *Rickettsia* detection in *Spodoptera litura***

To detect the presence of *Rickettsia* in *S. litura*, the egg (10 eggs as one unit), 3^rd^ instar larva, and adults were individually homogenized in lysis buffer and DNA was extracted and amplified using the protocol described in Gottlieb *et al*. (2006). The primers used for *Rickettsia* detection were Rb-F (5’-GCTCAGAACGAACGCTATC-3’) and Rb-R (5’-GAAGGAAA GCATCTCTGC-3’) for the*16S rRNA* gene. The PCR procedure entailed pre-denaturation at 94 ^◦^C for 2 min followed by 32 cycles of 94 ^◦^C (1 min), 58 ^◦^C (45 s) and 72 ^◦^C (45 s). All PCRs were performed in a 25 *μ*l reaction volume that included 2.5 mM MgCl_2_, 200 mM of each dNTP, 1 *μ*M of each primer and 1 unit DNA Taq polymerase (Invitrogen, Guangzhou, China). PCR amplified products were visualized on a 1% agarose gel containing Gold-View colourant. *Portiera aleyrodidarum* DNA was used as a positive control and ddH_2_O was used as a negative control to eliminate potential confounding variables. PCR and electrophoresis results revealed that the *S. litura* used in our current study was *Rickettsia* negative.

**2. Primers and protocol of *Rickettsia* PCR detection in the tomato plants infected with *Rickettsia* positive *Bemisia tabaci* AsiaII7**

**(1) Table S1. PCR primers for *Rickettsia* detection in both tomato leaf and root.**

| Primer | Sequence |
| --- | --- |
| *gltA* | F:5’-TCCTATGGCTATTATGCTTG-3’ |
|  | R:5’-CCTACTGTTCTTGCTGTGG-3’ |
| *16S-Rb* | F:5’-GCTCAGAACGAACGCTATC-3’ |
|  | R:5’-GAAGGAAAGCATCTCTGC-3’ |
| *Pgt*  (nested PCR) | F1:5’-AGGTTTAGGCTAGTCTACACG-3’ |
|  | R1:5’-GTCTACGCACGATTGATG-3’ |
|  | F2:5’-ACTCATGAAATTATCGGCACAG-3’ |
|  | R2:5’-GCATGAATTTGGCACTTAAGC-3’ |

**(2) PCR Protocol**

All PCRs were performed in a 25 *μ*l reaction volume that included 2.5 mM MgCl_2_, 200 mM of each dNTP, 1 *μ*M of each primer and 1 unit DNA Taq polymerase (Invitrogen, Guangzhou, China). PCR procedures entailed pre-denaturation at 94 ^◦^C for 2 min followed by 32 cycles of 94 ^◦^C (1 min), 55 ^◦^C for *gltA* gene/58 ^◦^C for 16S *Rb* gene/52 ^◦^C for *Pgt* gene (45 s), and 72 ^◦^C (45 s). PCR amplified products were visualized on a 1% agarose gel containing Gold-View colorant. When bands with the expected size were visible on the gels, 20 *μ*l volumes of PCR products were sent to Beijing Genomics Institute (BGI) for sequencing. *Portiera aleyrodidarum* DNA was used as a positive control and ddH_2_O was used as a negative control to eliminate potential confounding variables.

**3. Primers and protocol for q-PCR quantification of representative DEGs**

**(1) Table S2. Primers for q-PCR quantification.**

| Gene name | Primer sequences |
| --- | --- |
| *WRKY70* | F: 5’-TTCTGCTCTGAGTTGCATCG- 3’  R: 5’-ACTGTCCATGCCTCAGCATC- 3’ |
| *PR-1* | F: 5’-CGACACCGTAGCTGCCTATG- 3’  R: 5’-GCTGTAATCGTAGTCGGCCTTC- 3’ |
| *AOC* | F: 5’-ATCGCTACGAGGCCATCTTC-3’  R: 5’-TGGTGGAGCCTAACCTGACC-3’ |
| *LOX* | F: 5’-GCACATTGAGACCATTGGCGATTG-3’  R: 5’-AGCACCAGTGGCATACCAAGTTG-3’ |
| *PI-II* | F: 5’-CCCGTTCAGGAGGAAAGACG-3’  R: 5’-GGGTCACATTCCAGGGTACG-3’ |
| *JAZ1* | F: 5’-AATATGGATTCCGGCAAGGT-3’  R: 5’-TCATAGTAGTTGTGGTGGTAGTAGCTG-3’ |
| *VRP* | F: 5’-GGCAAGATGGAACCACAACACAAG-3’  R: 5’-TCCAAGGCAATCACATGACCAGAG-3’ |
| *TGA2.1* | F: 5’-CTTTCACAAGGTATGGAGGCG-3’  R: 5’-CGGGCTGATTGTCTGGTTG-3’ |
| *Rubisco* | F: 5’-CCTGATTTGTCTGACGAGCA-3’  R: 5’-GCACCCAAACATAGGCAACT-3’ |
| *TYLCV* | F: 5’-GAAGCGACCAGGCGATATAA-3’  R: 5’-GGAACATCAGGGCTTCGATA-3’ |
| *PaLCuCNV* | F: 5’-TAGTCATTTCCACTCCCGC-3’  R: 5’-TGATTGTCATACTTCGCAGC-3’ |

**(2) q-PCR Protocol**

RNA from the plant was extracted using RNA rep pure plant kit (Tiangen, Beijing). DNase was added to remove DNA contamination. The cDNA were synthesized using Prime ScriptTM RT reagent kit with gDNA Eraser (Takara, Japan). Amplifications were performed using Thunderbird SYBR Green PCR mix (TOYOBO, Osaka, Japan) and 5 p mol of each primer. The cycling conditions were: 5 min activation at 95 °C, 40 cycles of 30 s at 95 °C, 30 s at 55 °C and finally 30 s at 72 °C. A non-template negative control was included for each primer set to check for primer dimers and contamination.

**4. Disease assessment of *V. dahlia* on different treatment plants**

The disease symptoms caused by *V. dahliae* were classified following the method of Markakis *et al.* (2016). Disease incidences of *V. dahlia* on undamaged, *R-* and *R+* tomato plants were estimated as the percentage of infected plants. The disease severity index of each plant was based on an arbitrary scale from 0-4 grade of disease incidence: the infected leaves/total leaves ×100%, grade 0=0-20%, grade 1=21-40%, grade 2=41-60%, grade 3=61-80%, grade 4=81-100%. The percentage of disease index was calculated from the disease rating by the formula:

Disease severity index (%) = [∑ (grade rating no. × no. of plants in the rating)/total no. of plants× highest rating] × 100 %. Experiments were repeated three times.

**References:**

Gottlieb Y, Ghanim M, Chiel E, Dan E, Zchori-Fein E et al. 2006. Identification and localization of a *Rickettsia* sp. in *Bemisia tabaci* (Homoptera: Aleyrodidae). Appl Environ Microbiol 72: 3646-52.

Gupta G, Rani S, Birah A, Raghuraman M. 2005. Improved artificial diet for mass rearing of the tobacco caterpillar, *Spodoptera litura* (Lepidoptera: Noctuidae). Inter J Trop Insect Sci 25: 55-58.

Liu Y, Fan ZY, An X, Shi PQ, Ahmed MZ, Qiu BL. 2020. A single-pair method to screen *Rickettsia* infected and uninfected whitefly *Bemisia tabaci* populations. J Microbiol Meth 168: 105797.

Markakis EA, Tjamos SE, Antoniou PP, Paplomatas EJ, Tjamos EC. 2016. Biological control of *Verticillium wilt* of olive by *Paenibacillus alvei*, strain K165. Biocontrol 61: 293-303.

Qiu BL, Chen YP, Liu L, Peng WL, Li XX. 2009. Identiﬁcation of three major *Bemisia tabaci* biotypes in China based on morphological and DNA polymorphisms. Prog Nat Sci 19: 713–718.

**Supplementary Results**

**The RNA-Seq analysis of *Rickettsia* positive, *Rickettsia* negative and control tomato plants**

An average of 23,788,192, 23,900,603, and 23,865,519 clean reads were generated, after filtering out adapter sequences and low-quality reads (tags with the unknown nucleotide “N”), from the 3 libraries (Ctrl, *R-*, and *R+*). Among the data of the three clean read libraries, the mean of 90.17% to 94.67% of the clean reads were successfully mapped to the reference genome (Table S3).

Compared to Ctrl, there were 7157 [4047 (56.55%) upregulated and 3110 (43.45%) downregulated], and 6538 [3360 (51.39%) upregulated and 3178 (48.60%) downregulated] DEGs that were altered in *R-* and *R+*, respectively. Compared to *R-*, there were 2583 DEGs [1029 (39.84%) upregulated and 1554 (60.16%) downregulated] that were altered in *R+* (Table S4). A Venn diagram analysis showed that 962 DEGs were commonly expressed among all the treatments. (Figure S5).

Gene ontology (GO) analysis showed that the distribution of gene functions for these genes was assigned to three GO categories: biological process, cellular component, and molecular function (Figure S6). KEGG signaling pathway analyses revealed that biosynthesis of secondary metabolites, plant-pathogen interaction and plant hormone signal transduction were the top three responsive pathways (Figure S7).

**Table** **S3**. **Overview of RNA sequencing dataset.**

| Treatment^§^ | Sample | Clean reads | Clean date rate | Gene mapping ratio (%) | Genome mapping  ratio (%) |
| --- | --- | --- | --- | --- | --- |
| Ctrl | 1 | 23,734,301 | 98.76% | 79.62% | 91.02 % |
|  | 2 | 23,964,283 | 99.67% | 85.60% | 96.69 % |
|  | 3 | 23,665,992 | 98.53% | 84.44% | 96.29 % |
|  | Mean | 23,788,192 | 98.99% | 83.22% | 94.67% |
| *R-* | 1 | 23,924,781 | 99.65% | 81.56% | 93.88% |
|  | 2 | 23,876,134 | 98.53% | 72.52% | 84.61% |
|  | 3 | 23,900,894 | 99.53% | 79.71% | 92.01% |
|  | Mean | 23,900,603 | 99.24% | 77.93% | 90.17% |
| *R+* | 1 | 23,681,577 | 98.54% | 77.66% | 89.38% |
|  | 2 | 23,982,351 | 99.79% | 78.65% | 91.10% |
|  | 3 | 23,932,629 | 99.62% | 79.52% | 91.68% |
|  | Mean | 23,865,519 | 99.32% | 78.61% | 90.72% |

^§^Ctrl, control plants; *R-*, plants were pre-infested with *R-* whiteflies; *R+*, plants were pre-infested with *R+* whiteflies.

**Table S4. The number of upregulated and downregulated genes (differentially expressed genes, DEGs) of tomato plants pre-infested with whiteflies for 7 days.**

| Group^§^ | Number of DEGs | | |
| --- | --- | --- | --- |
|  | Down | Up | Total |
| Ctrl vs. *R-* | 3110 | 4047 | 7157 |
| Ctrl vs. *R+* | 3178 | 3360 | 6538 |
| *R-* vs. *R+* | 1554 | 1029 | 2583 |

^§^Ctrl, control plants; *R-*, plants were pre-infested with *R-* whiteflies; *R+*, plants were pre-infested with *R+* whiteflies.

**Supplementary Figures**

**
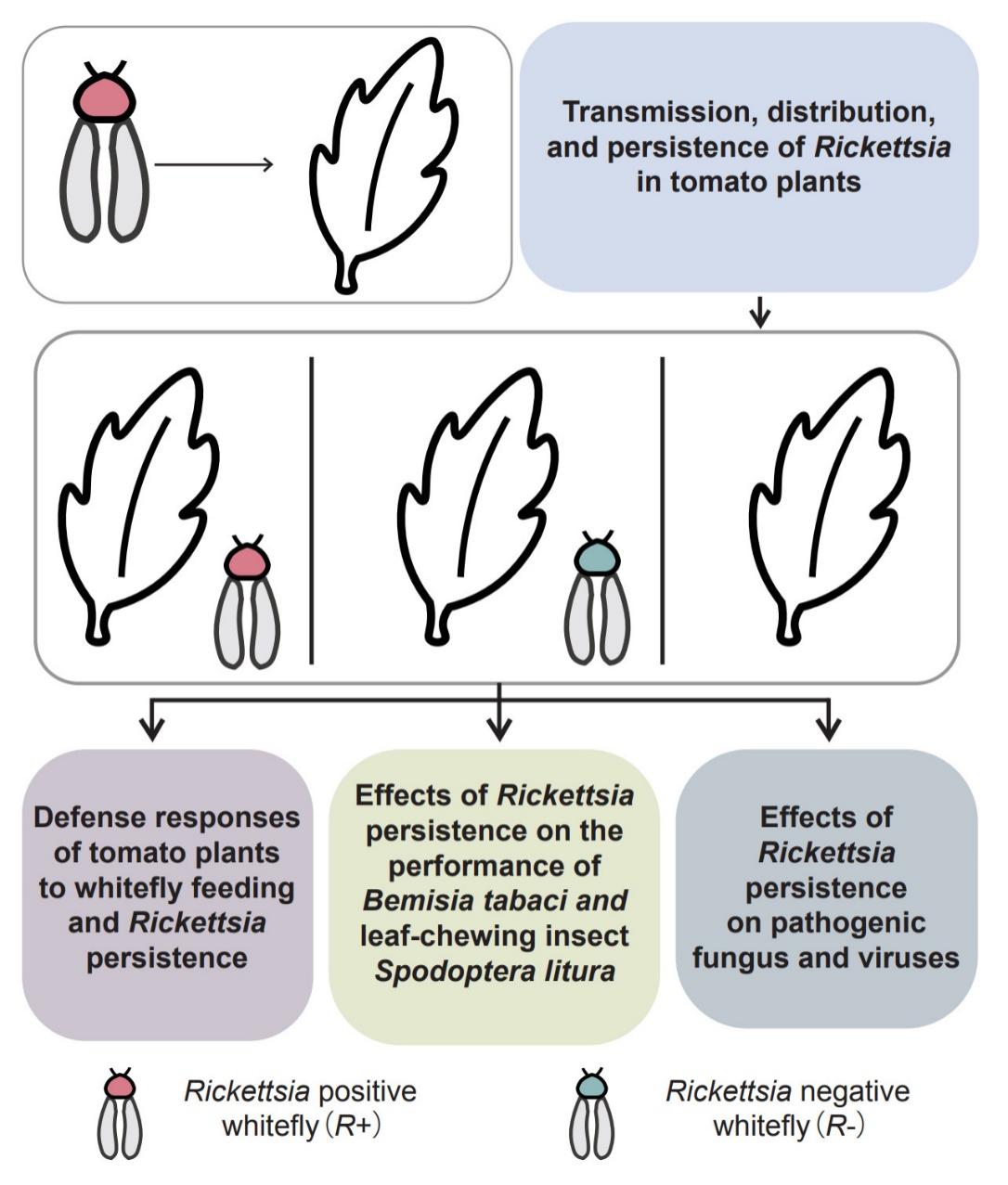
**

**Figure S1. Experimental design.**

**
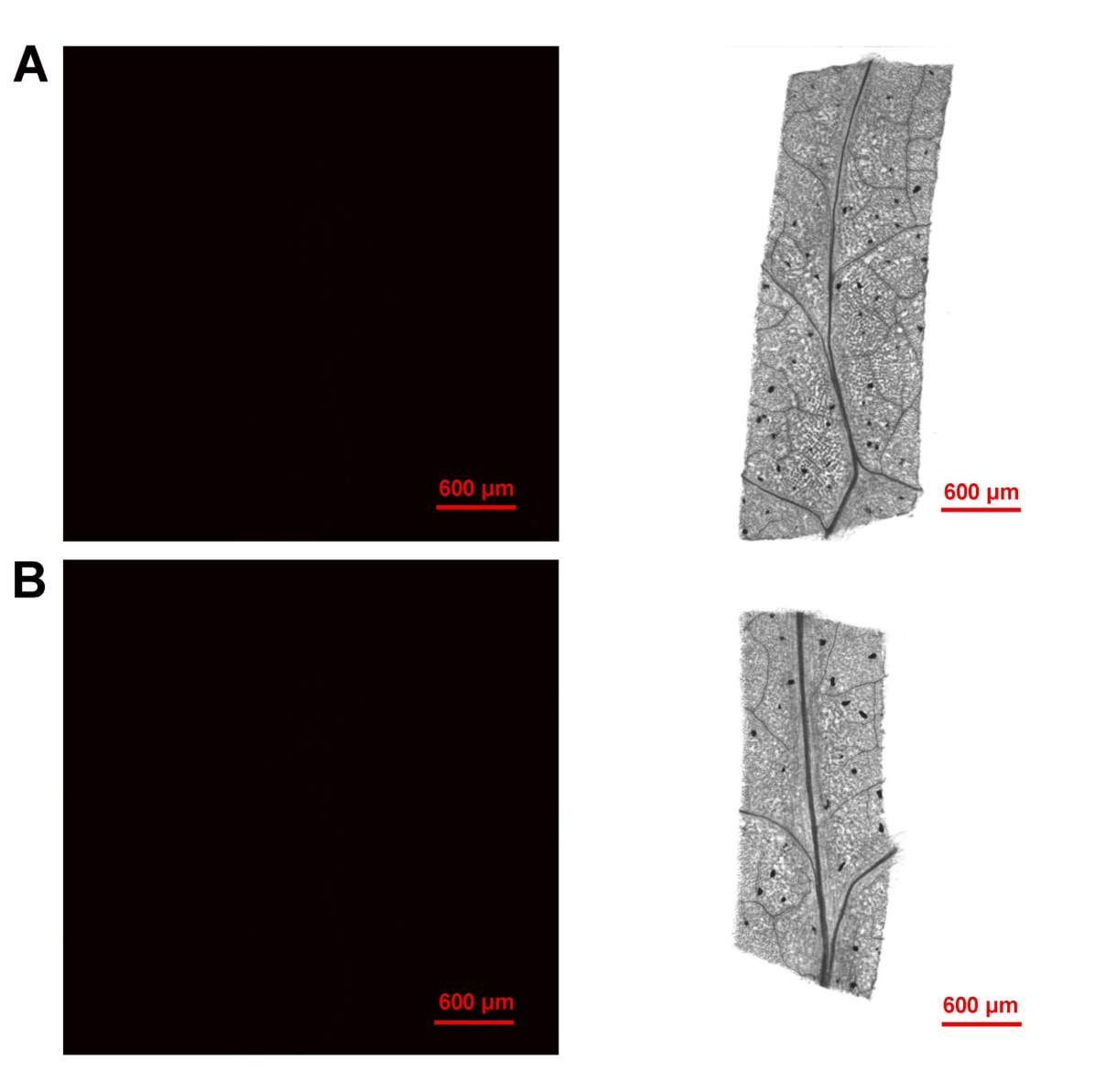
**

**Figure S2. Negative control of *Rickettsia* transmission into tomato leaves with fluorescence in situ hybridization.**

A: healthy tomato leaf infested with *R-* whiteflies; B: tomato leaf infested with *R+* whiteflies but without symbiont-specific *16S rRNA* probe hybridization. Left panels: fluorescence in dark field; right panels: fluorescence in bright field. No fluorescence of *Rickettsia* was visualized in these negative controls.


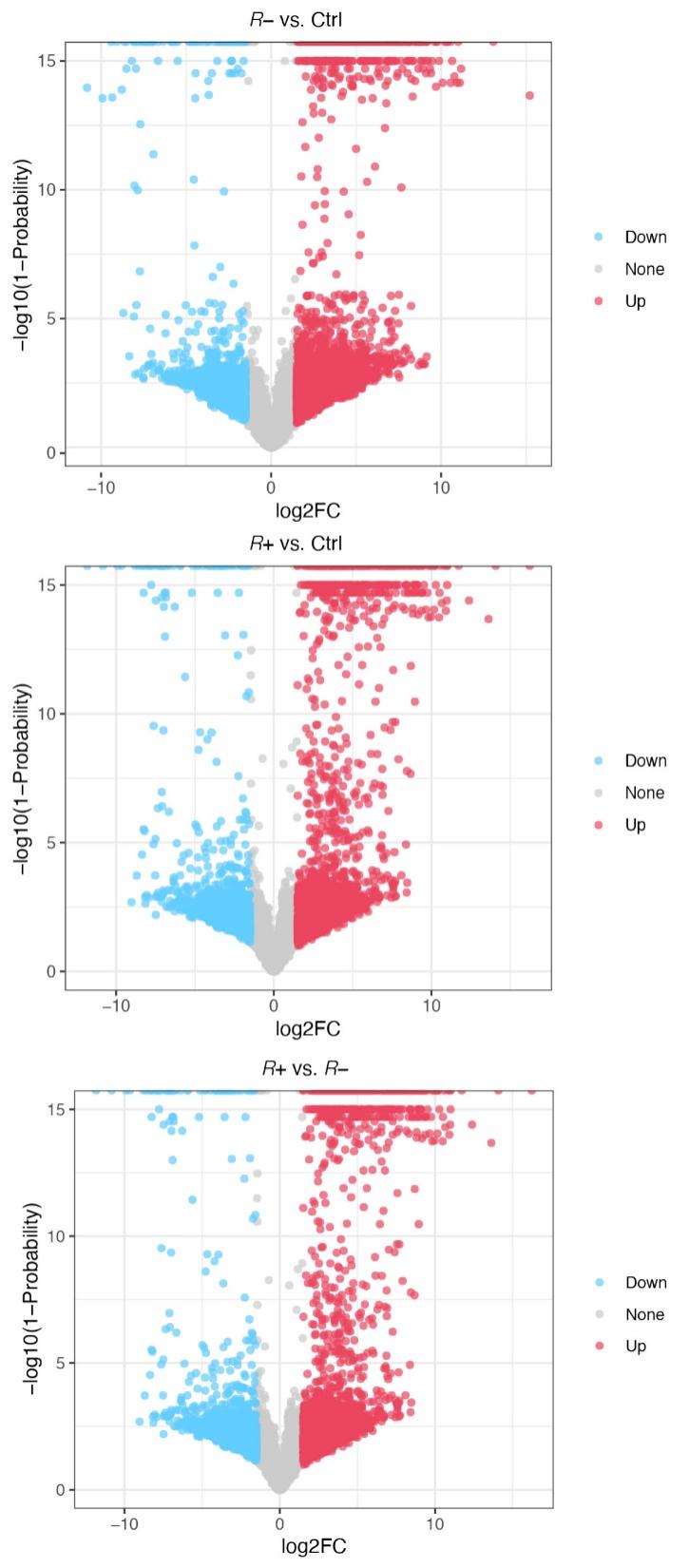


**Figure S3. Volcano plots of differentially expressed genes (DEGs).**

Ctrl, control plants; *R*-, plants were pre-infested with *R*- whiteflies; *R*+, plants were pre-infested with *R*+ whiteflies.


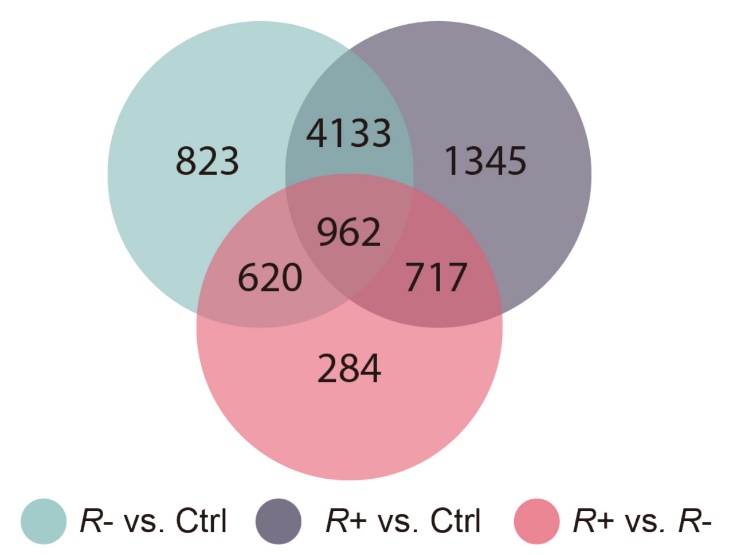


**Figure S4**. **A Venn diagram of differentially expressed genes (DEGs) in tomato plants pre-infested with whiteflies for 7 days**. Ctrl, control plants; *R-*, plants were pre-infested with *R-* whiteflies; *R+*, plants were pre-infested with *R+* whiteflies.


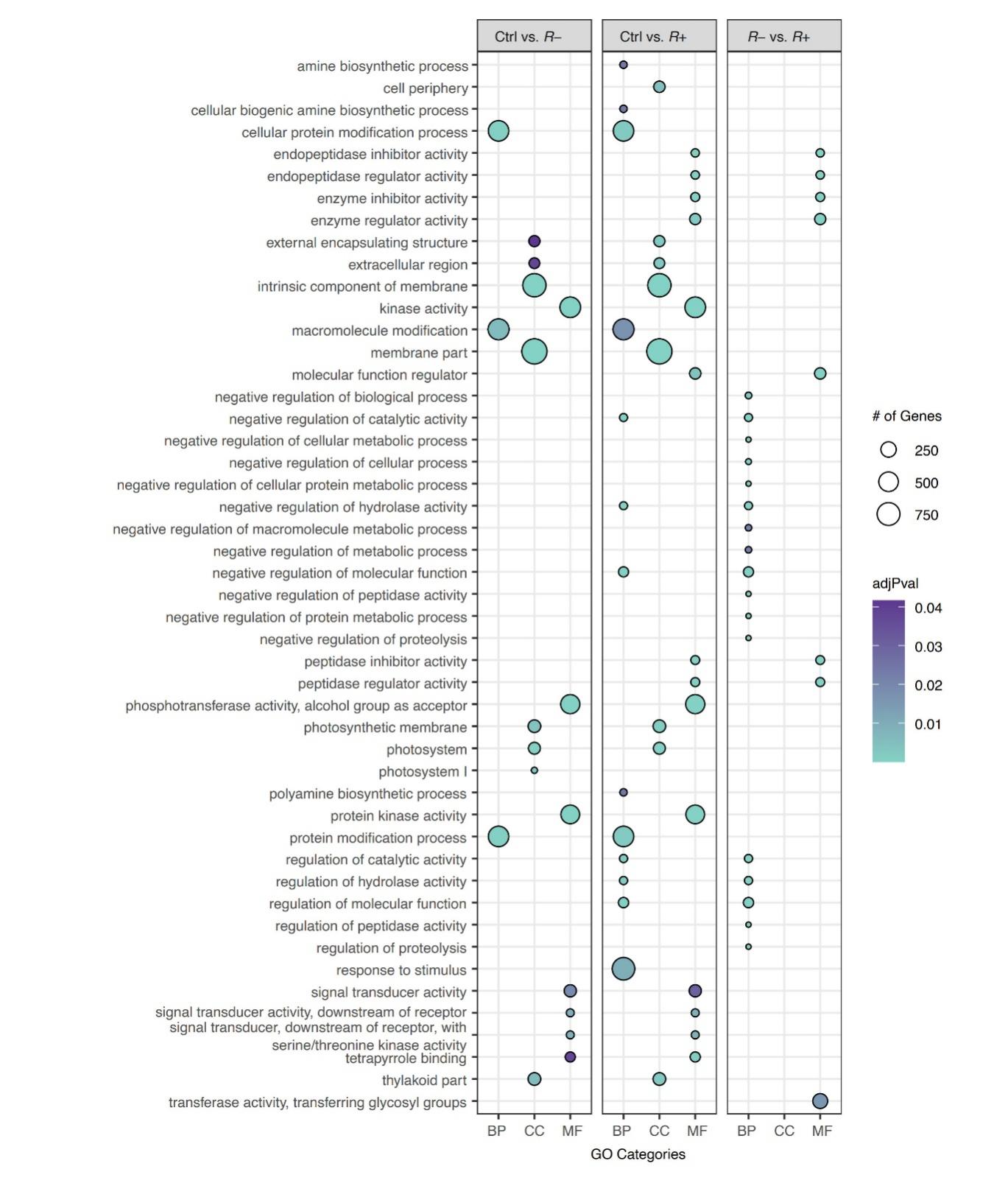


**Figure S5.** **Summary of gene ontology (GO) annotations**. X-axis shows the GO terms. BP: biological process, CC: cellular component, MF: molecular function. Ctrl: control plants, *R-*: plants were pre-infested with *R-* whiteflies, *R+*: plants were pre-infested with *R+* whiteflies.


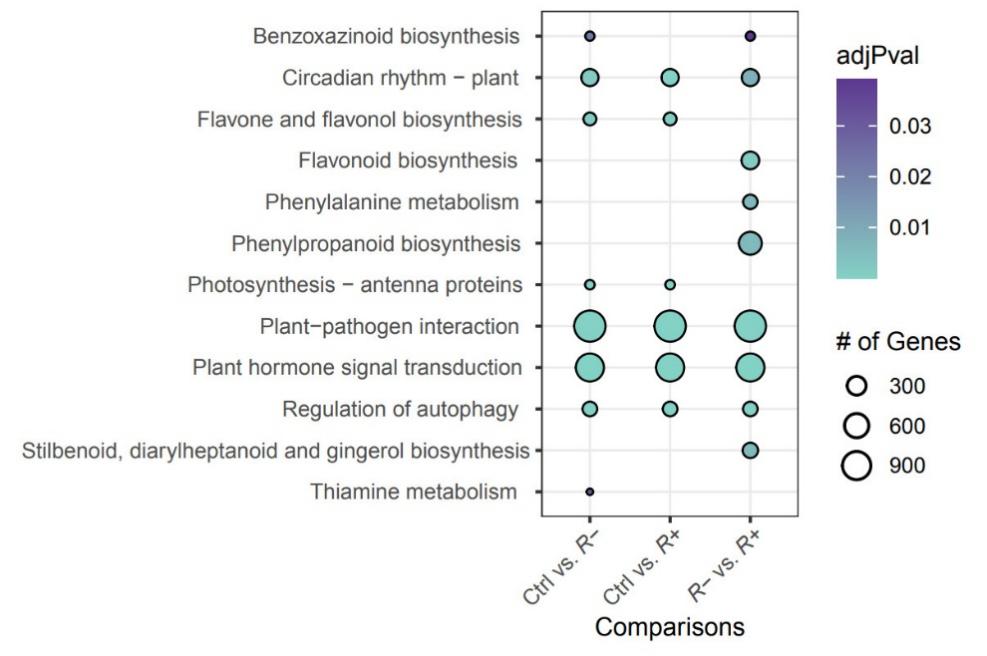


**Figure S6.** **KEGG pathway annotation classification of differentially expressed genes (DEGs) of tomato plants pre-infested with whiteflies for 7 days**.

X-axis shows the KEGG classification. Ctrl, control plants; *R-*, plants were pre-infested with *R-* whiteflies; *R+*, plants were pre-infested with *R+* whiteflies.

**
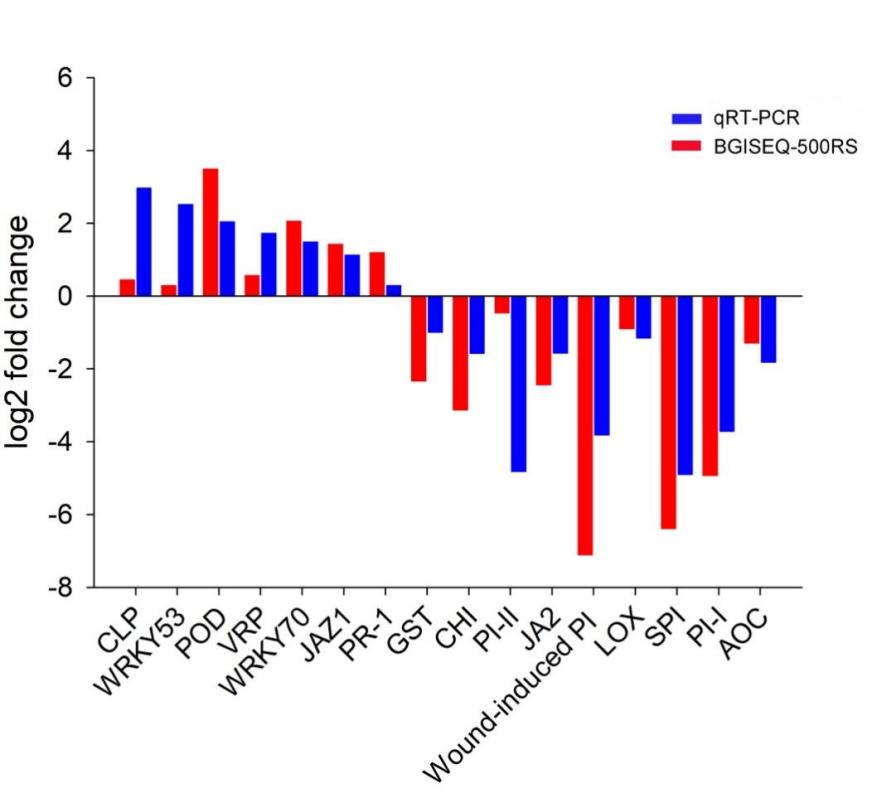
**

**Figure S7. Relative expression validation of differentially expressed genes by q-PCR.**

Red: the gene expression levels [log2 fold change (*R+* plant/*R-* plant)] detected by Beijing Genomics institute (BGI-Shenzhen, China) with the BGISEQ-500RS platform. Blue: the expression level [log2 fold change (*R+* plant/*R-* plant)] detected by q-PCR. The *RuBisCo* gene (ribulose-bisphosphate carboxylase) of the tomato plant was used as a reference gene, and the 2^−ΔΔCT^ method was implemented. SA responsive genes: *WRKY 53*, WRKY transcription factor 53 (Solyc08g008280.3.1); *VRP*, Verticillium wilt disease resistance protein 2 (Solyc09g005080.1.1); *WRKY70*, WRKY transcription factor 70 (Solyc03g095770.3.1); *PR-1*, pathogenesis-related protein-1 protein (Solyc09g007010.1.1). JA responsive genes: *JAZ1*, jasmonate ZIM-domain protein 1 (Solyc12g009220.2.1); *CHI*, chitinase (Solyc04g072000.3.1); *PI-II*, Proteinase inhibitor II (Solyc03g020070.3.1); *JA2*, Jasmonic acid 2 (Solyc12g013620.2.1); Wound-induced *PI*, Wound-induced proteinase inhibitor 1 (Solyc09g084470.3.1); *LOX*, Lipoxygenase (Solyc01g009680.3.1); *PI-1*, Proteinase inhibitor I (Solyc09g089505.1.1); *AOC*, allene oxide cyclase (Solyc02g085730.3.1). Other genes: *CLP*, Calmodulin-like protein (Solyc11g071740.2.1); *POD*, Peroxidase (Solyc11g018777.1.1); *GST*, Glutathione S-transferase (Solyc12g056250.2.1); *SPI*, Type I serine protease inhibitor (Solyc09g084480.3.1).
